# First Observation of MTH FR 678 C-A (Ala222Ala) Single Nucleotide Polymorphism

**DOI:** 10.5152/tjh.2011.45

**Published:** 2012-06-15

**Authors:** Yonca Eğin, Nejat Akar

**Affiliations:** 1 Ankara University, School of Medicine, Department of Pediatric Molecular Genetics, Ankara, Turkey

## TO THE EDITOR

The enzyme methylenetetrahydrofolate reductase (MTHFR) plays a key role in folate metabolism. MTHFR catalyses NADPH-linked reduction of N5,10-methylenetetrahydrofolate to N5-methylene tetrahydrofolate. The MTHFR gene exhibits 2 common genetic polymorphisms (C677T and A1298C). The MTHFR 677 C to T substitution converts alanine to a valine residue, resulting in a thermolabile enzyme [1]. A reduction in MTHFR activity leads to an elevated plasma homocysteine level, which has been implicated as a risk factor for vascular and thromboembolic disease [2,3,4]. 

Real-time polymerase chain reaction (RT-PCR) facilitates quantification of polymorphic DNA regions and genotyping of single nucleotide polymorphisms (SNPs), including MTHFR C677T base substitution. The system is optimized to detect the difference in the Tm of the mutant and the wild-type allele using commercially available primers and hybridization probes [5]. Recently, MTHFR C685G (Iso225Val) and G679A (Asp223Asn) mutations other than the common MTHFR C677T were reported based on different melting points and RT-PCR [6], with an allele frequency of 1 in 3000-4000 samples [5]. 

A male patient’s DNA was isolated using a MagnaPure automatic isolation system (Roche Diagnostics, Indianapolis, USA). Genotyping of the MTHFR C677T polymorphism was performed via RT-PCR and fluorescence melting curve detection analysis using a LightCycler system (Roche Diagnostics, Manheim, Germany). Written informed consent was provided by the patient. 

The PCR product was purified using a Wizard SV PCR clean-up system (Promega, Madison, WI, USA), and sequenced using a CEQ Dye Terminator Cycle sequencing kit and a CSQ 8000 genetic analysis system (Beckman Coulter, Brea, CA, USA). Nucleotide changes were confirmed via a new PCR reaction, followed by sequencing analysis The patient had an unusual RT-PCR analysis melting point profile. The patient’s DNA was further analyzed via DNA sequencing, which showed a nucleotide change at 678 C>A that does not lead to an amino acid change (Ala- Ala). This polymorphism was not previously reported. Retrospectively, 4100 samples from our laboratory’s archives were analyzed, but none of these samples had an MTHFR 678C-A change. 

Research has shown that worldwide there is great variation in the prevalence of the MTHFR 677C-T mutation [2,7,8]. Several laboratory techniques are used to detect MTHFR C677T; the most common and reliable technique is melting point analysis, which can differentiate variants that lie within the region of the target probe. Differences in the base composition and nucleotide position affect Tm in wild-type and target mutations. Variants with Tm shifts of 2-5 °C are easily visualized during experiments, as was the case of MTHFR and other thrombophilia genes [5,9,10].

In the present study we reported a new heterozygous MTHFR gene polymorphism (678 C>A), with an unusual melting point profile. Although rare, the finding of MTHFR 678C>A polymorphism indicates that this might be technically important, as it may lead to erroneous reporting. 

**Conflict of Interest Statement **

The authors of this paper have no conflicts of interest, including specific financial interests, relationships, and/ or affiliations relevant to the subject matter or materials included.
